# Supplementary material for: Beyond Treatment Decisions: The Predictive Value of Comprehensive Geriatric Assessment in Older Cancer Patients
Source: Cancers (Basel). 2025 Jul 28;17(15):2489. doi: 10.3390/cancers17152489 (PMC12346502; doi:10.3390/cancers17152489)
Supplement: Supplementary file 1 [file cancers-17-02489-s001.zip › cancers-3741547-supplementary.pdf]

Table S1. Elements of the Comprehensive Geriatric Assessment by Cohabitation Status

Variable	With spouse/partner (n=929)	Alone (n=1264)	With family (n=4310)	p-value
**MNA**	23.00±12.58	22.50±10.15	22.50±5.22	0.87
**ADL**	6.00±1.28	6.00±2.67	6.00±1.32	<0.001
**IADL**	7.00±2.94	8.00±1.91	7.00±2.13	<0.001
**GDS**	4.00±2.94	4.00±2.97	4.00±2.77	<0.001
**MMSE**	26.30±3.72	27.00±7.75	27.40±2.73	0.13
**CIRS-CI**	1.27±1.49	1.24±1.37	1.12±1.27	0.005

Abbreviations: MNA: Mini Nutritional Assessment; ADL: Activities of Daily Living; IADL: Instrumental Activities of Daily Living; MMSE: Mini-Mental State Examination; GDS: Geriatric Depression Scale; CIRS-CI: Cumulative Illness Rating Scale-Comorbidity Index.

Table S2. Elements of the Comprehensive Geriatric Assessment by Cohabitation Status, sorted by Age Groups.

Variable	Age<77				Age≥77			
	With spouse/partner (n=444)	Alone (n=423)	With family (n=1980)	p-value	With spouse/partner (n=483)	Alone (n=837)	With family (n=2325)	p-value
**MNA**	23.66±15.34	22.87±12.52	22.53±9.03	0.26	22.65±9.26	22.35±8.77	24.33±6.68	0.81
**ADL**	5.59±1.05	5.84±4.22	5.50±1.18	<0.001	5.10±1.43	5.39±1.20	5.15±1.45	<0.001
**IADL**	6.64±1.87	7.03±1.67	6.73±1.88	<0.001	5.50±2.40	6.44±1.99	4.48±2.78	<0.001
**GDS**	3.74±2.74	4.46±3.06	4.33±2.76	<0.001	4.14±3.11	4.50±2.92	4.48±2.78	0.08
**MMSE**	26.12±3.11	27.47±12.58	29.27±6.15	0.62	24.91±4.13	26.24±3.31	27.33±4.07	<0.001
**CIRS-**	1.08±1.36	1.04±1.24	1.02±1.95	0.78	1.45±1.58	1.34±1.42	1.21±1.32	0.005

**CI**	

Abbreviations: MNA: Mini Nutritional Assessment; ADL: Activities of Daily Living; IADL: Instrumental Activities of Daily Living; MMSE: Mini-Mental State Examination; GDS: Geriatric Depression Scale; CIRS-CI: Cumulative Illness Rating Scale-Comorbidity Index.

Figure S1. Kaplan-Meier curves of overall 4-year survival stratified by GDS scores (below 10 vs. above 10 points), sorted by age groups.

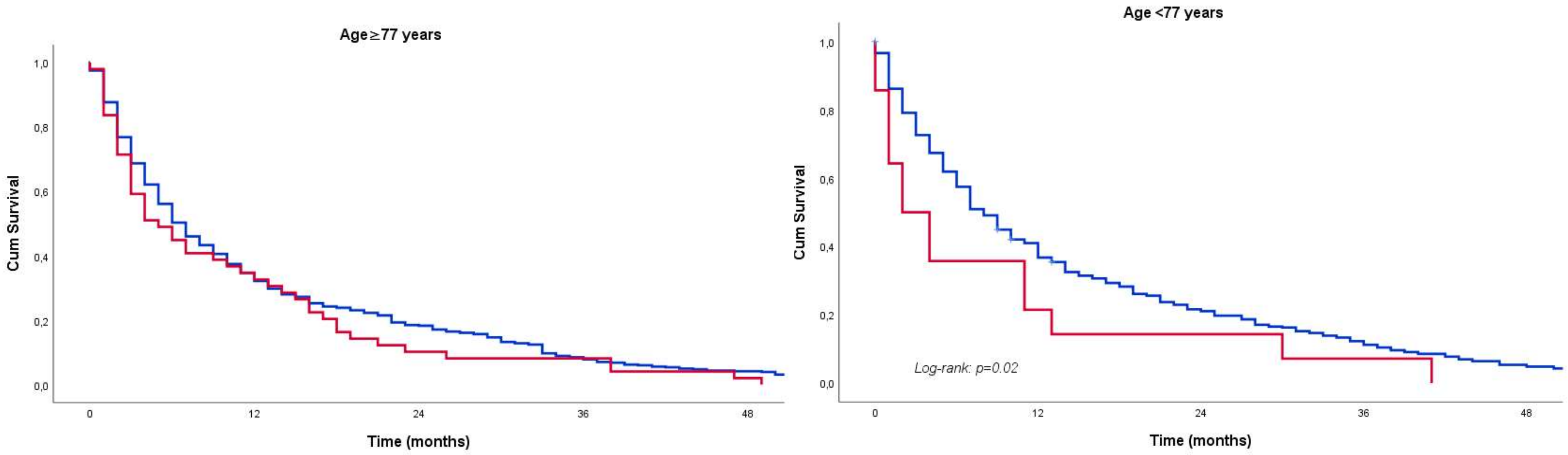

Table S3. Prognostic values for overall 4-years survival of CGA elements: Cox proportional-hazard models, sorted by age groups.

AGE<77 YEARS								
Variable	***Unadjusted analysis***				***Adjusted analysis***			
	HR	***95%IC***		***p-value***	***HR***	***95%IC***		***p-value***
		Lower	***Upper***			***Lower***	***Upper***	
Cohabitative status								
Alone	***0.60***	***0.45***	***0.79***	***<0.001***	0.93	0.61	1.42	0.74
With other family members/carers	***0.59***	***0.38***	***0.90***	***0.01***	***1.42***	***1.05***	***1.93***	***0.03***
**Multidimensional evaluation**								
MNA	0.99	0.98	1.07	0.59	0.99	0.98	1.01	0.37
IADL	***0.89***	***0.81***	***0.98***	***0.02***	***0.89***	***0.79***	***0.97***	***0.008***
MMSE	***1.08***	***1.02***	***1.14***	***0.007***	***1.08***	***1.02***	***1.14***	***0.008***
GDS	***1.07***	***1.01***	***1.13***	***0.02***	***1.07***	***1.01***	***1.13***	***0.02***
CIRS-CI	0.95	0.85	1.07	0.41	0.94	0.84	1.06	0.35
**Total n. drugs**	1.05	0.89	1.10	0.11	1.06	0.98	1.17	0.06
**Frail**	0.92	0.60	1.41	0.70	1.16	0.68	1.96	0.58

AGE≥77 YEARS								
Variable	***Unadjusted analysis***				***Adjusted analysis***			
	HR	***95%IC***		***p-value***	***HR***	***95%IC***		***p-value***
		Lower	***Upper***			***Lower***	***Upper***	
Cohabitative status								
Alone	0.51	0.07	3.78	0.51	1.42	0.83	1.57	0.42
With other family members/carers	0.59	0.08	4.37	0.60	***1.87***	***1.38***	***2.53***	***<0.001***
**Multidimensional evaluation**								
MNA	***0.96***	***0.93***	***0.98***	***0.002***	***0.95***	***0.92***	***0.99***	***0.007***

IADL	***0.92***	***0.86***	***0.98***	***0.01***	***0.92***	***0.86***	***0.99***	***0.04***
MMSE	0.98	0.95	1.02	0.33	0.98	0.95	1.03	0.51
GDS	0.99	0.95	1.04	0.81	1.01	0.96	1.05	0.84
CIRS-CI	***1.02***	***1.08***	***1.19***	***0.04***	0.91	0.84	1.01	0.06
**Total n. drugs**	0.98	0.94	1.03	0.41	0.98	0.94	1.03	0.42
**Frail**	1.09	0.82	1.46	0.55	0.89	0.61	1.32	0.58

Abbreviations: MNA: Mini Nutritional Assessment; IADL: Instrumental Activities of Daily Living; MMSE: Mini-Mental State Examination; GDS: Geriatric Depression Scale; CIRS-CI: Cumulative Illness Rating Scale-Comorbidity Index.

Analyses are adjusted for age, gender, cancer treatment and site, and metastasis.
